# Nontoxic pyrite iron sulfide nanocrystals as second electron acceptor in PTB7:PC_71_BM-based organic photovoltaic cells

**DOI:** 10.3762/bjnano.10.216

**Published:** 2019-11-14

**Authors:** Olivia Amargós-Reyes, José-Luis Maldonado, Omar Martínez-Alvarez, María-Elena Nicho, José Santos-Cruz, Juan Nicasio-Collazo, Irving Caballero-Quintana, Concepción Arenas-Arrocena

**Affiliations:** 1 Research Group of Optical Properties of Materials (GPOM), Centro de Investigaciones en Óptica A. C., León, Guanajuato, 37150, México; 2 Departamento de Ingeniería en Energía, Universidad Politécnica de Guanajuato, Guanajuato, 38483, México; 3 Centro de Investigación en Ingeniería y Ciencias Aplicadas, UAEM, Morelos, 62210, México; 4 Facultad de Química, Energía-Materiales, Universidad Autónoma de Querétaro, Querétaro, 76010, México; 5 Escuela Nacional de Estudios Superiores Unidad León; Universidad Nacional Autónoma de México, León, Guanajuato, 37684, México

**Keywords:** iron disulfide, nanoparticles, organic photovoltaic cells (OPVs), PTB7, pyrite

## Abstract

Herein, we report the synthesis of nontoxic pyrite iron sulfide (FeS_2_) nanocrystals (NCs) using a two-pot method. Moreover, we study the influence of these NCs incorporated into the PTB7:PC_71_BM active layer of bulk-heterojunction ternary organic photovoltaic (OPV) cells. The OPV devices are fabricated with the direct configuration glass/ITO/PEDOT:PSS/PTB7:PC_71_BM:FeS_2_/PFN/FM. The Field’s metal (FM) is a eutectic alloy composed of 32.5% Bi, 51% In and 16.5% Sn by weight that melts at 62 °C. It is deposited on the active layer/PFN under atmospheric conditions. Ternary active layers are prepared by adding small amounts of the semiconducting FeS_2_ NCs at different weight ratios of 0.0, 0.25, 0.5, and 1.0 wt % with respect to the electron donor PTB7. With respect to the reference device (without FeS_2_), a 21% increase in the power conversion efficiency (PCE) is observed for OPVs with 0.5 wt % FeS_2_, such that the PCE of the OPVs is enhanced from 5.69 to 6.47%. According to the Kruskal–Wallis and Mann–Whitney statistical tests, all OPV devices follow the same trend.

## Introduction

Iron disulfide (FeS_2_) is a natural earth-abundant and nontoxic material with possible applications in lithium batteries, transistors or photovoltaic (PV) devices [1–2]. According to the analysis carried out by Wadia et al. [3], among 23 semiconducting materials, FeS_2_ is the best candidate for the development of large-scale solar cells at low cost (<2 × 10^−6^ ¢/W). Furthermore, FeS_2_ exhibits excellent optoelectronic properties such as a band gap of 0.8 to 1.38 eV [4–8], a high optical absorption coefficient (2 × 10^5^ cm^−1^) [4], high carrier mobility (2 to 80 cm^2^/Vs) [4,9] and a large charge carrier lifetime (200 ps) [10]. Therefore, FeS_2_ nanoparticles (NPs) can be a good alternative for PV applications [11]. Nanostructures of FeS_2_ have been used as counter electrodes in dye-sensitized solar cells (DSSCs) [9,12–13], as electron acceptors or donors in inorganic or hybrid solar cells [10,14–17] and as second electron acceptors in organic photovoltaic cells (OPVs) [18]. An iron pyrite thin film used as a counter electrode showed a conversion efficiency (8%) similar to that of a Pt counter electrode in DSSC [9], which could be due to the high catalytic activity of pyrite. When the film is doped with ethanedithiol (EDT), the conversion efficiency is increased by about 20% as compared to the pure pyrite thin film [12]. Also, FeS_2_ NPs of 30 nm size inside the CdS/CdTe inorganic heterojunction improved the conversion efficiency of the PV device by up to 8% [19]. Moreover, FeS_2_ has also been used in perovskite solar cells as a hole transport layer (HTL) to reduce the fabrication cost, reaching efficiencies of up to 11.2% [20].

On the other hand, OPVs have been widely studied due to their advantages such as low-cost, easy fabrication, flexibility, low environmental impact and semitransparency [21–23]. Nowadays, OPVs have already achieved a power conversion efficiency (PCE) of 17.3% in the tandem architecture [24]. Also, commercial OPV panels have obtained a PCE of ≈2% at a size of 2.52 × 0.52 m [25]. The bulk-heterojunction (BHJ) approach is the most promising configuration to enhance the interpenetrated interface area in OPVs. In previous works, OPVs based on poly(3-hexylthiophene) (P3HT) or poly(thieno[3,4-*b*]thiophene-*co*-benzodithiophene) (PTB7) blended with [6,6]-phenyl C71-butyric acid methyl ester (PC_71_BM) have shown PCEs of >2% and >7%, respectively, with standard electrodes (Al, Ag) deposited through a high-vacuum evaporation process [26–29]. In our previous work [30–33], we used the eutectic Field’s metal (FM) as an alternative top electrode, which we attached by free vacuum deposition. The FM is composed of 32.5% Bi, 51% In and 16.5% Sn and is characterized by a melting point of around 62 °C. It was deposited on the active layer/PFN by drop casting (or doctor blade) at low temperature (≈95 °C) and atmospheric conditions [30,32–34]. The performance of the OPVs depends on the misalignment of the energy levels of each component, as well as on the organic compounds and the architecture used, on the type of solvents, the deposition technique of the active layer, the annealing conditions and the thickness and morphology of the devices [32,35]. Spin-coating is one of the most widely used techniques for active layer deposition that provides a small active area film with a low root-mean-squared roughness of about 1–3 nm [36].

It has been proved that, with the incorporation of a third component in the OPV active layer, the harvesting solar energy is usually enhanced as well as the charge transport and the charge collection behavior at the electrodes, and in some cases, also the lifetime stability is increased. However, such effects depend on the type of the third compound and its concentration in the active layer [37–38]. Therefore, several strategies have been applied to increase the efficiency of ternary OPVs. The photovoltaic parameters were significantly improved with the use of a second donor [39–40] or acceptor (fullerene or nonfullerene) [41–42]. For instance, isocyanate-treated graphene has been used to dope the active layer based on P3HT, increasing the conversion efficiency by 59% compared to the undoped devices [43]. Also, solution processable functionalized graphene (SPFG) was incorporated as a third component in PTB7:PC_71_BM active layers obtaining a PCE increment of 22% with respect to the reference devices [21]. Cheng et al. used ICBA (di[1,4]methanonaphthaleno[1,2:2',3';56,60:2'',3''][5,6]fullerene-C60-Ih, 1',1'',4',4''-tetrahydro-) to provide more routes for charge transfer at the PTB7:PC_71_BM interface, improving the average efficiency from 7.23 to 8.13% [41]. Wang et al. [44], used the nonfullerene acceptor molecule ITIC (2,2′-[[6,6,12,12-tetrakis(4-hexylphenyl)-6,12-dihydrodithieno[2,3-d:2′,3′-d′]-s-indaceno[1,2-b:5,6-b′]dithiophene-2,8-diyl]bis[methylidyne(3-oxo-1H-indene-2,1(3H)-diylidene)]]bis[propanedinitrile]) with PBDB-T (poly[[4,8-bis[5-(2-ethylhexyl)-2-thienyl]benzo[1,2-*b*:4,5-*b*′]dithiophene-2,6-diyl]-2,5-thiophenediyl[5,7-bis(2-ethylhexyl)-4,8-dioxo-4*H*,8*H*-benzo[1,2-*c*:4,5-*c*′]dithiophene-1,3-diyl]]) and PC_71_BM to form efficient electron-transport pathways, achieving an enhanced PCE of 10.2% as compared to 9.2 and 8.1% for the binary PBDB-T:ITIC and PBDB-T:PC_71_BM devices. The addition of magnetic oxide nanoparticles to the OPV P3HT:PC_70_BM active layer has improved the lifetime and the stability of these devices with an efficiency of ≈3% [45]. On the other hand, 5 wt % Fe_3_O_4_ NPs doped into the P3HT:PCBM blend increased the PCE from 1.09 to 2.22% [46]. Lin et al. [18], reported an increment of the PCE from 2.08 to 2.3% with the incorporation of small amounts of FeS_2_ nanocrystals (NCs) (0.3 wt %) into the P3HT:PC_71_BM active layer. Moreover, Khan et al. [47], added 20 wt % FeS_2_ quantum dots (of ≈5 nm size) and obtained a high PCE of 3.62% compared to the reference device with a PCE of 2.32%. Hence, FeS_2_ NCs have widely been used to enhance the efficiency of solar cells by improving dissociation and charge transport.

**Figure 1:**
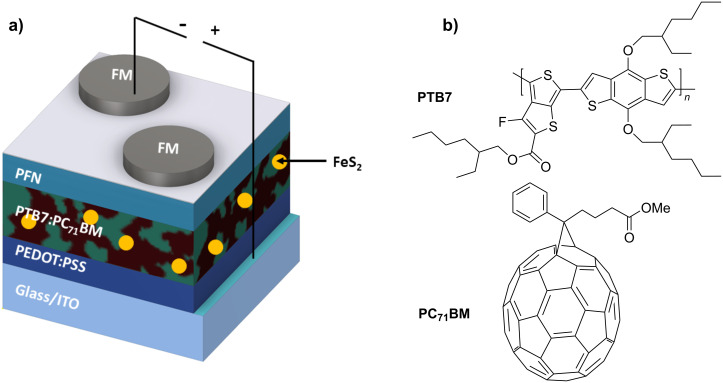
a) OPV structure. FM: Field’s metal (Bi, In, Sn); b) Chemical structure of PTB7 and PC_71_BM.

In our previous work [48], we reported the noncytotoxicity of cube-like FeS_2_ NCs by studying mouse fibroblast cells at different reaction temperatures. In this work, semispherical pyrite NCs are synthesized and added at different concentrations as second electron acceptors into the PTB7:PC_71_BM active layer of the OPVs that are fabricated with the direct configuration glass/ITO/PEDOT:PSS/PTB7:PC_71_BM:FeS_2_/PFN/FM, where PFN is poly[(9,9-bis(3'-(*N*,*N*-dimethylamino)propyl)-2,7-ﬂuorene)-*alt*-2,7-(9,9-dioctylﬂuorene)] (see Figure 1a and the Experimental section). Figure 1b shows the chemical structure of PTB7 and PC_71_BM. We observe an improvement of the OPV performance by 21% using this nontoxic and low-cost iron pyrite (0.5 wt %) semiconductor material. The PCE of the OPV is enhanced from 5.69% to 6.47%. To the best of our knowledge, the addition of FeS_2_ NCs to a PTB7:PC_71_BM active layer has not been analyzed before.

## Results and Discussion

**Figure 2:**
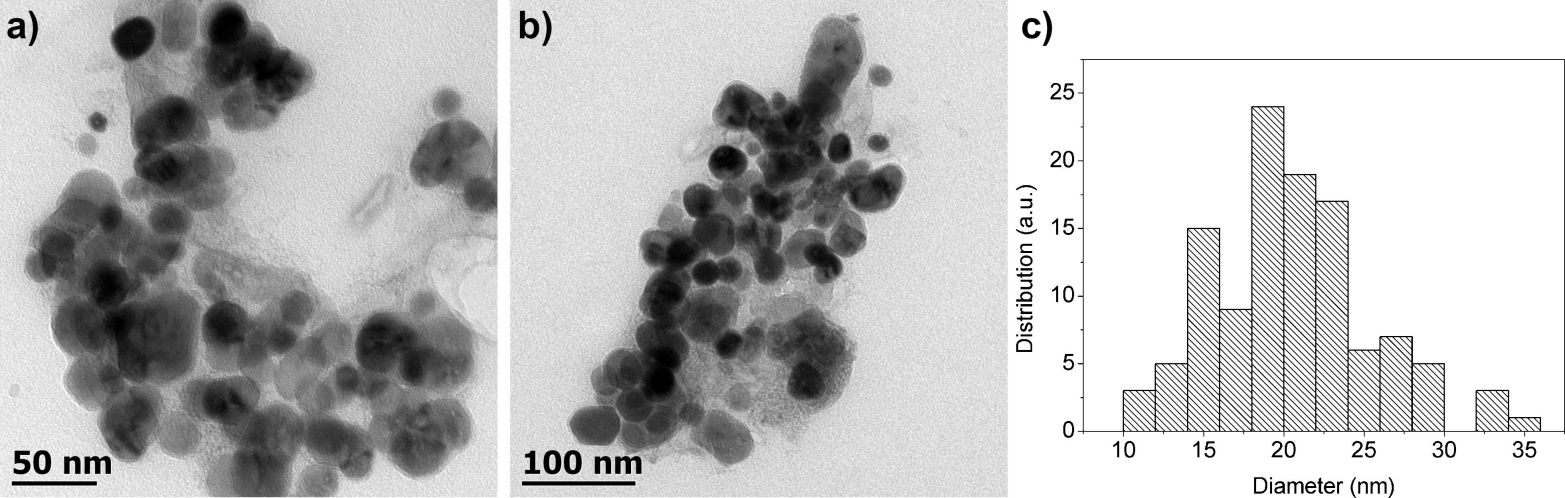
a) and b) TEM images of the FeS_2_ NCs at the 50 and 100 nm scale and c) size distribution of the NCs.

Semispherical FeS_2_ NCs with an average size of 20 ± 4 nm were observed using transmission electron microscopy (TEM) (Figure 2a and 2b). The size distribution of the NCs was determined with the Image J software (Figure 2c). The X-ray pattern of these NCs, reported in our previous work [48], showed peaks at 2θ = 28, 33, 37, 40.7, 47.5, 56, 61.5 and 64.5°, corresponding to the pyrite crystalline phase (pyrite JCPDS (Joint Committee on Powder Diffraction Standards) card), which is in good agreement with the reported cubic morphology [49].

**Figure 3:**
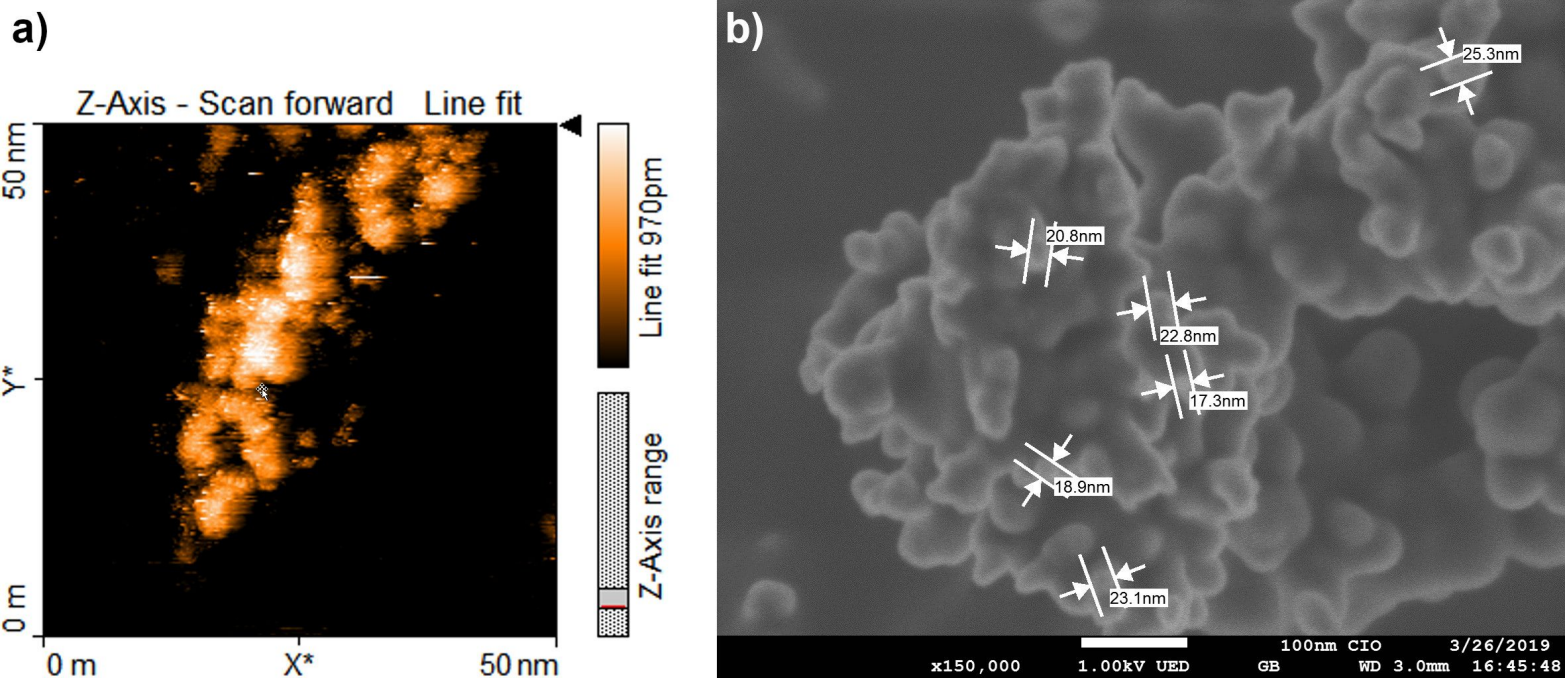
a) STM image of FeS_2_ deposited on HOPG substrate (thickness ≈20 nm) with 50 nm × 50 nm scan size and b) SEM image of FeS_2_ NCs (scale bar = 100 nm).

FeS_2_ thin films were analyzed using scanning tunneling microscopy (STM) to study their molecular ordering at the nanoscale level. Figure 3a shows the STM image. The scanned area (*A*), the tunneling current (*I*_t_) and the applied potential (*U*) are *A* = 50 nm × 50 nm, *I*_t_ = 500 pA and *U* = 450 mV. The scanning electron microscopy (SEM) image of agglomerated FeS_2_ NCs is shown in Figure 3b. The results verify that the sizes of the NCs lie within the nanoscale regime (about 15 to 25 nm).

**Figure 4:**
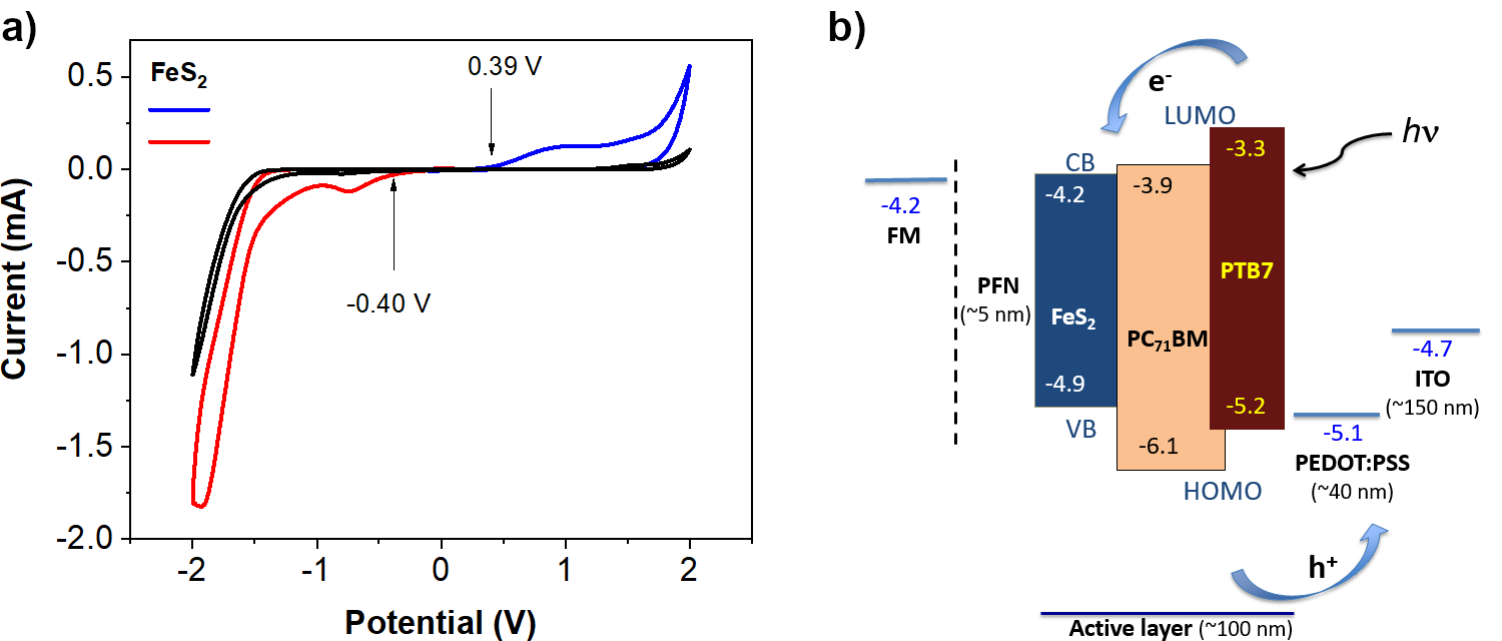
a) Cyclic voltammograms of ITO (reference, black) and FeS_2_ NCs in TBAPF_6_ 0.1 M at 100 mV s^−1^ (blue: anodic zone, red: cathodic zone). b) Flat energy diagram of the organic materials and FeS_2_ used for OPV fabrication [21].

The energy levels of the FeS_2_ NCs were characterized by cyclic voltammetry (CV), as shown in Figure 4a. The voltammograms show an oxidation peak, assigned to the ionization potential, at approximately +0.39 V and a reduction peak, assigned to the electron affinity, at about −0.40 V. In order to estimate the electrochemical band gap energy, the valence and conduction band energies (*E*_VB_ and *E*_CB_) were calculated using the following Equation 1 and Equation 2:

[1] $$E_{\text{VB}}=-\left(E_{\left[\text{onset}\text{,ox}\right]}-E_{\left[\frac{1}{2}\left(\text{Fc}\right)\right]}+4.8\right)\text{eV}$$

and

[2] $$E_{\text{CB}}=-\left(E_{\left[\text{onset}\text{,red}\right]}-E_{\left[\frac{1}{2}\left(\text{Fc}\right)\right]}+4.8\right)\text{eV}$$

where *E*_[onset,ox]_ and *E*_[onset,red]_ are the onset potentials of the oxidation and the reduction relative to ferrocene/ferrocenium (Fc^+^/Fc), *E*_[½(Fc)]_ is the half-wave ferrocene potential of 0.20 V, and the additional energy of 4.8 eV represents the difference to the vacuum level potential of the normal hydrogen electrode. Thus, we determine an *E*_VB_ of −4.99 eV and an *E*_CB_ of −4.20 eV, resulting in a reasonable band gap energy of 0.79 eV, which is in line with the range reported in the literature [4–8]. Figure 4b shows the energy diagram of FeS_2_ and the organic materials used for OPV fabrication. FeS_2_ as a second acceptor was successfully applied to improve the performance of the OPVs by the generation of additional charge carrier pathways and by the cascade-like shaping of the energy levels of the active layer materials (as shown in the diagram) [21]. Possibly, charges might travel through three different pathways: PTB7-FeS_2_ (free holes–electrons), PC_71_BM-FeS_2_ (free electrons) and PTB7-PC_71_BM (free holes–electrons) [28,41,43,50]. These characteristics could facilitate exciton dissociation, charge transport and collection processes, and thus increase the overall PCE value.

**Figure 5:**
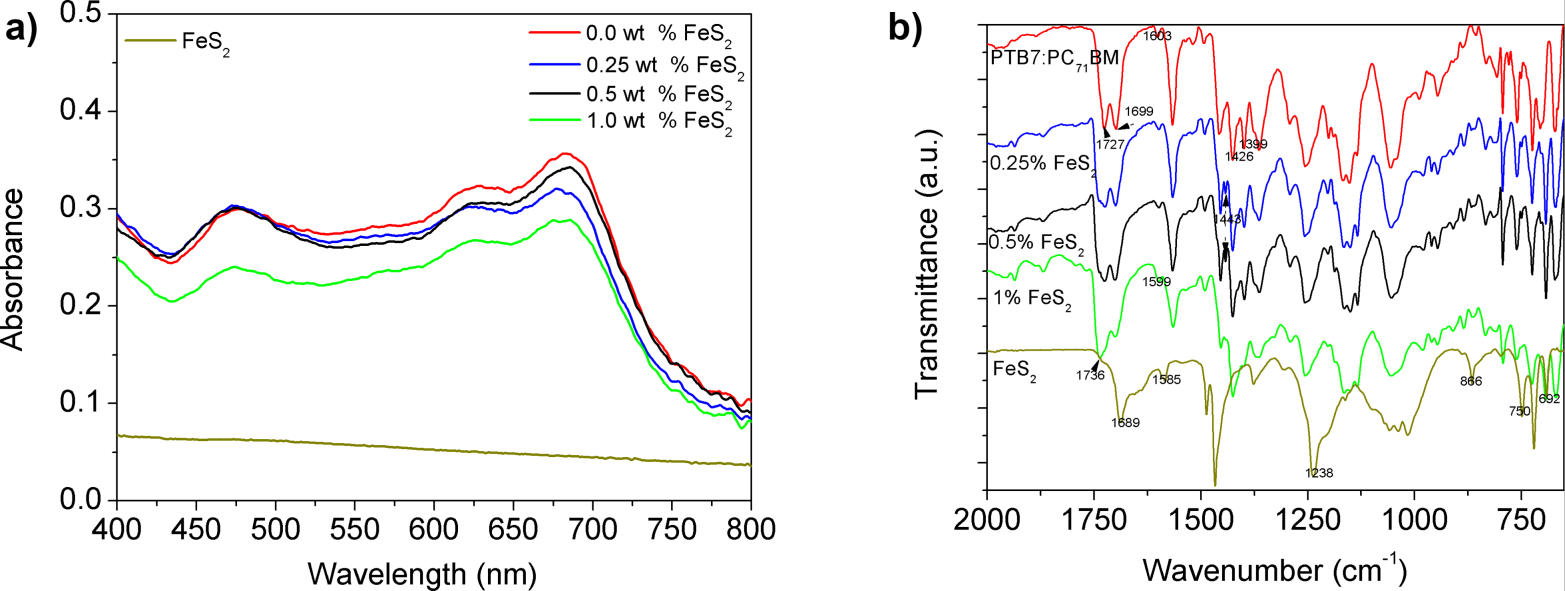
a) UV–vis absorption and b) FTIR spectra of the blend PTB7:PC_71_BM at different FeS_2_ concentrations: 0.0, 0.25, 0.5 and 1.0 wt %.

Figure 5a shows the UV–vis absorption spectra of the OPV active layer at different concentrations of FeS_2_ as well as that of the pure FeS_2_ film. The spectrum of the PTB7:PC_71_BM active layer presents a broad absorption in the visible range (300 to 750 nm). In all absorption spectra, PTB7 peaks appeared at 630 and 700 nm and PC_71_BM peaks were observed at 375 and 480 nm, as reported elsewhere [51]. The FeS_2_ NCs do not contribute to the absorption spectra and the light harvesting due to the low amount added to the PTB7:PC_71_BM active layer [18]. Yet, the difference in the absorption of the active layers could arise from slight differences in the sheet thicknesses, and some light dispersion is most likely due to the modified optical quality. Figure 5b shows the Fourier transform infrared (FTIR) spectra of the PTB7:PC_71_BM blend at different concentrations of FeS_2_. The band at 1727 cm^−1^, corresponding to the C=O stretching mode in PTB7:PC_71_BM [52], becomes ≈15 cm^−1^ wider upon the incorporation of FeS_2_. Specifically at 1 wt % of FeS_2_, the band at 1736 cm^−1^ becomes larger. This band intensity increase could suggest some intermolecular interaction between the O atom of the carbonyl groups of PTB7 and PC_71_BM with the Fe atoms of the FeS_2_ NCs. In addition, upon the incorporation of FeS_2_ into the PTB7:PC_71_BM mixture, the 1603 cm^−1^ band was observed to shift red by ≈4 cm^−1^ to 1599 cm^−1^, and a small peak appeared at 1443 cm^−1^. At higher FeS_2_ concentration, the spectrum is modified in the region from 1475 to 1385 cm^−1^. The peaks at 866 cm^−1^ and 692 cm^−1^ belong to the bands characteristic of FeS_2_ [16,49]. The FeS_2_ band at 1689 cm^−1^ is not observed for the PTB7:PC_71_BM:FeS_2_ blends because of the wide PTB7:PC_71_BM band located in that wavenumber range.

**Figure 6:**
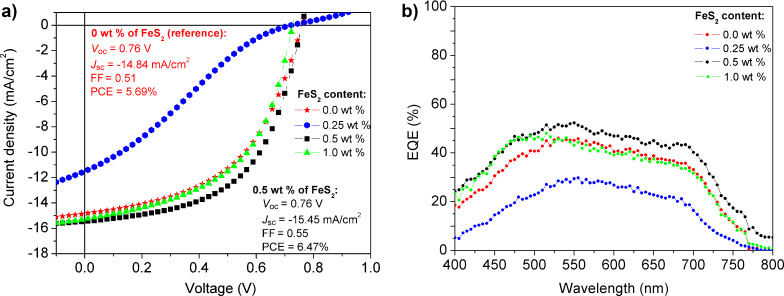
a) *J*–*V* curves and b) EQE of PTB7:PC_71_BM-based devices with different concentrations of FeS_2_: 0.0, 0.25, 0.5 and 1.0 wt %.

Figure 6a shows the current density *J* as a function of the voltage *V* (*J*–*V* plots) of the OPVs with 0, 0.25, 0.5 and 1 wt % of FeS_2_ as third component in the ternary active layer. For the reference device containing no FeS_2_ (0 wt %), a PCE of 5.69% is determined. For the OPV with 0.5 wt % of FeS_2_, a PCE of 6.47% is reached, which is mainly due to an increment of the short circuit current density (*J*_sc_) and fill factor (FF) while the open circuit voltage (*V*_oc_) remains nearly the same.. For each FeS_2_ concentration, three different OPV sets were fabricated and tested, and the results followed the same trend. Figure 6a shows the best values measured. The external quantum efficiency (EQE) plots of the OPVs are shown in Figure 6b. The EQE curves indicate that the photocurrent is generated mainly in the 400–750 nm range, in correlation with the absorption of the PTB7:PC_71_BM blend. The EQE of devices with 0.5 wt % of FeS_2_ is higher than that of the reference OPV cells, which could be attributed to a better charge separation (exciton dissociation enhancement) as well as enhanced carrier transport and collection in the corresponding devices [21,28,43,50]. In other words, a reduction of the exciton recombination can take place while additional electron charge pathways are established in the active layer. Furthermore, a better charge balance (free holes/electrons) develops, and thus, the overall OPV performance is improved.

The average PV parameters of the fabricated OPVs with different amounts of FeS_2_ are shown in Table 1. The best average efficiency value of 6.02% is observed for the device with 0.5 wt % of FeS_2_ added to the PTB7:PC_71_BM. On the contrary, for the OPV with 0.25 wt % of FeS_2_, we observe a significant decrement of the PV parameters (PCE = 1.57%) compared to the reference OPV (average PCE = 4.98%).

**Table 1:** Average PV parameters of three OPV sets with different FeS_2_ content in the active layer. Concerning the PCE, the numbers in parenthesis are the best values obtained.

FeS_2_ (wt %)	Thickness (nm)	*V*_oc_ (V)	FF	*J*_sc_ (mA/cm^2^)	PCE (%)	Roughness (nm)

0.0	101	0.73	0.47	14.48	4.98 (5.69)	1.1
0.25	109	0.64	0.23	11.15	1.57 (2.09)	1.4
0.5	110	0.77	0.52	15.31	6.02 (6.47)	1.7
1.0	106	0.71	0.50	15.21	5.48 (5.97)	3.2

The Kruskal–Wallis test [53] confirms that the observed differences in the electrical parameters (*V*_oc_, FF, *J*_sc_ and PCE) between the OPVs are indeed due to the different concentrations of the FeS_2_ NCs (Table S1 and S2, Supporting Information File 1). Also, the Mann–Whitney test [54] was applied to compare the different OPVs (Table S2). The obtained results collected in Supporting Information File 1, Table S2 confirm that the PCE of the reference device without FeS_2_ is significantly different from that of the OPVs with 0.5 wt % FeS_2_. However, the average *V*_oc_ value of the OPVs with 0.5 wt % FeS_2_ is not significant with respect to that of the reference devices (Supporting Information File 1, Table S2). The average value is however higher compared to that of the other OPVs (Table 1). Moreover, there is no significant difference in the electrical parameters of the OPVs with 0.5% and 1.0% (Table S2).

As shown in Figure 6a, the OPVs with 0.25 wt % of FeS_2_ exhibit an S-shaped *J*–*V* curve, that could arise from charge recombination or accumulation resulting from poor charge transport between the OPV interfaces, and/or it could be a sign of the poor quality of the BHJ active layer [55–58]. This S-curve shows a linear zone (low fill factor) attributed to a large series resistance, which could be due to the poor and irregular distribution (accommodation) of isolated FeS_2_ NCs in the active blend at this low concentration (0.25 wt %) inducing a reduction of *J*_sc_ and FF and thus of the overall PCE. In addition, the PCE starts to decrease for OPVs with 1 wt % of FeS_2_ (5.48%), which could be due to the presence of larger FeS_2_ agglomerates in the active layer.

**Figure 7:**
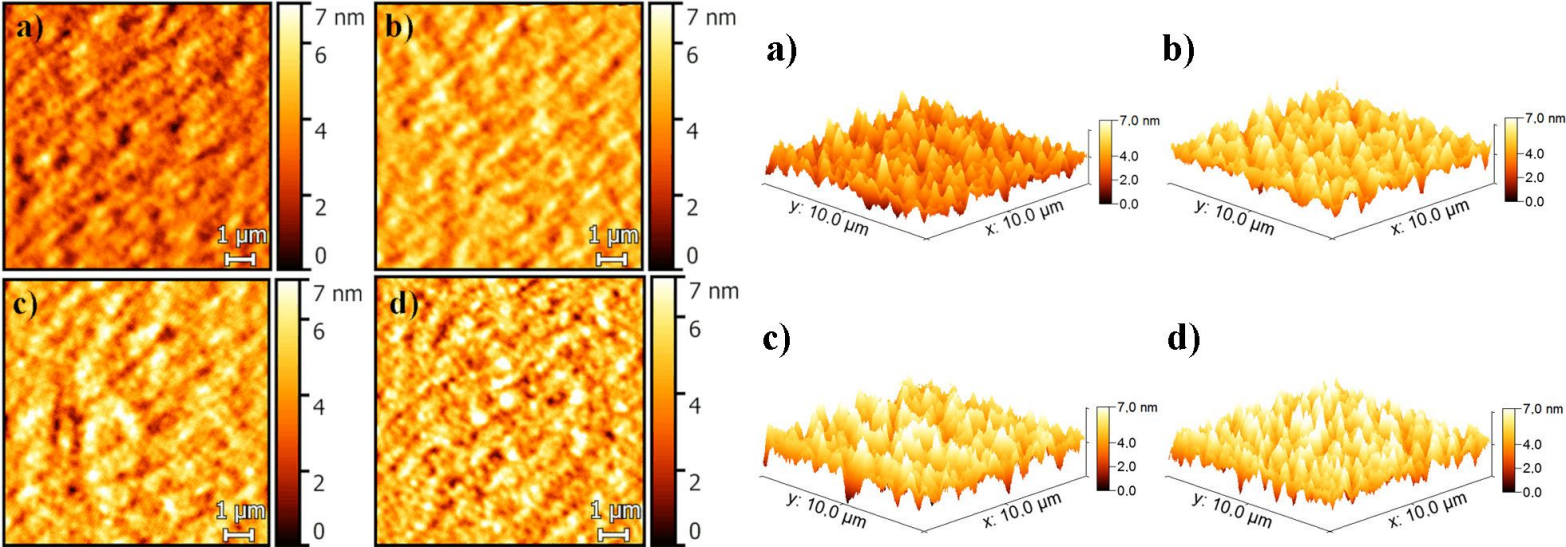
2D (left) and 3D (right) AFM images of the OPVs with different concentrations of FeS_2_ (a) 0.0 wt %, b) 0.25 wt %, c) 0.5 wt % and d) 1.0 wt %) recorded in noncontact mode.

Figure 7 shows the 2D (left) and 3D (right) AFM images of the OPVs with different concentrations of FeS_2_ recorded in the noncontact mode. The roughness of the OPV surface is increased gradually as the FeS_2_ concentration increases (Table 1 and Figure 7), such that traps for the charge carriers could occur and the leakage current could increase. Because of the FeS_2_ agglomerates, the OPV parameters tend to decrease, free charges cannot be efficiently extracted. This effect is most prominent for the OPV cells with 1% of FeS_2_ (Figure 7 and Supporting Information File 1, Figure S2d).

Figure S2 in Supporting Information File 1 shows a SEM image of the OPV layers cross-section. We observe thicknesses of each layer that acceptably correlate with the sheet thicknesses determined by the AFM measurement in contact mode, namely ITO ≈197 nm, PEDOT:PSS ≈40 nm and PTB7:PC_71_BM active layer ≈113 nm.

Figure S3(a–d) in Supporting Information File 1 shows the SEM images of the OPV active layers with different concentrations of FeS_2_. The observed granular structure of the ITO layer (Figure S3a–d, Supporting Information File 1) is due to the high voltage applied in the measurement (15 kV). In comparison, the ITO layer cannot be distinguished in the PEDOT:PSS image (Figure S3e, Supporting Information File 1) taken at low voltage (1 kV). These SEM images are complementary to the AFM images shown in Figure 7. Some NCs are highlighted by red circles, however, it is not trivial to unambiguously identify the NCs because they are immersed in the polymer matrix. Comparing the NC distribution over the active layers with 0.25 wt % and 0.5 wt % of FeS_2_, Figure S3b and Figure S3c in Supporting Information File 1 indicate that most of the NCs are rather isolated at lower concentration. In contrast, for the OPV with 1 wt % FeS_2_ NCs, agglomerates are observed.

**Figure 8:**
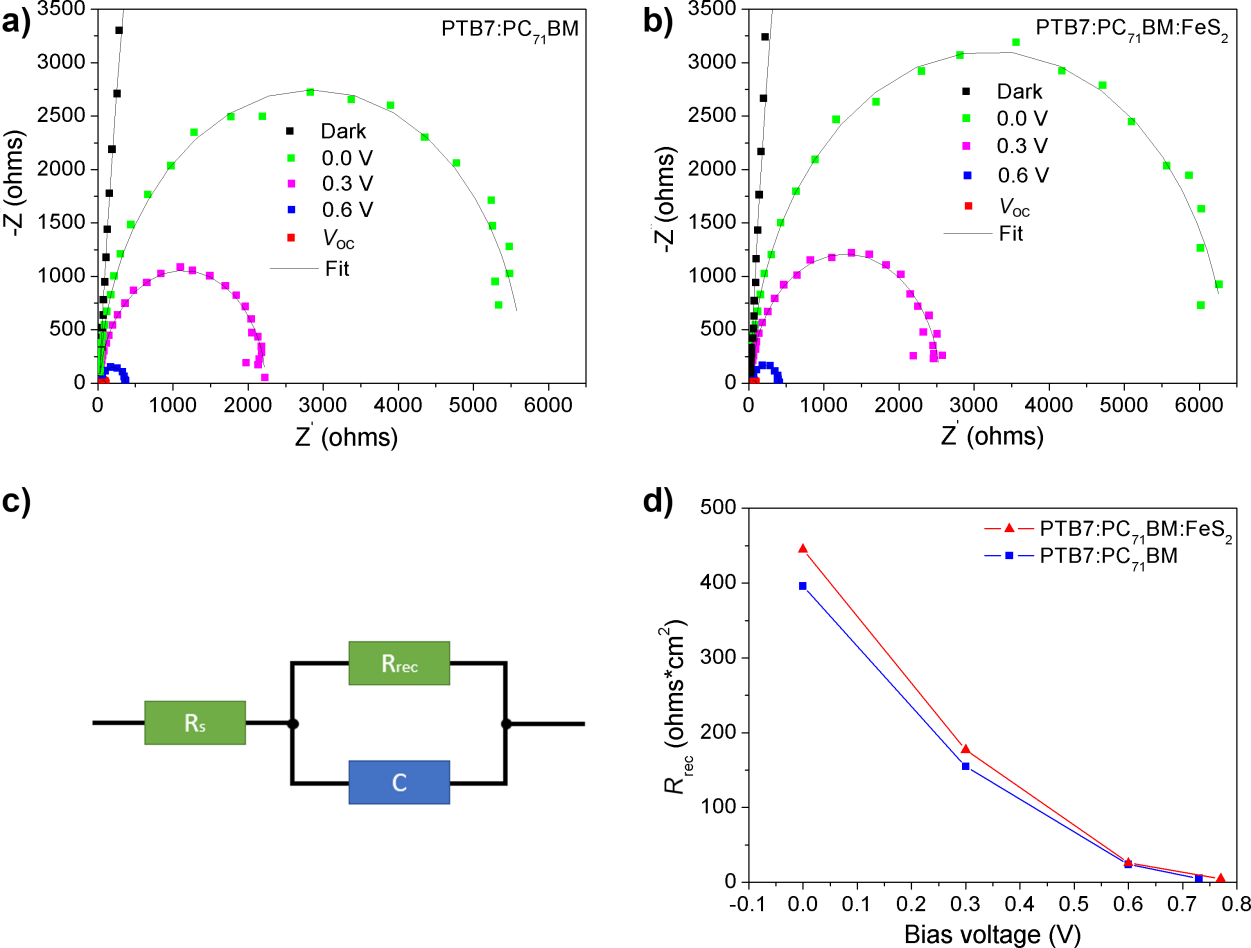
Impedance spectroscopy measurements (filled color squares) and simulations (black lines) of a) PTB7:PC_71_BM and b) PTB7:PC_71_BM:FeS_2_ with 0.5 wt % of FeS_2_; c) equivalent circuit used for the IS simulations; d) *R*_rec_ values vs bias voltage of PTB7:PC_71_BM (blue) and PTB7:PC_71_BM:FeS_2_ (with 0.5 wt % of FeS_2_) (red).

To study the charge carrier transport and recombination in the devices based on PTB7:PC_71_BM and PTB7:PC_71_BM:FeS_2_ with 0.5 wt % FeS_2_, impedance spectroscopy (IS) was conducted. IS is an important technique for monitoring charge carrier transport and recombination processes in solar cells [59]. These measurements provide information about the different factors limiting solar cell efficiency: charge storage, carrier lifetimes, recombination and resistivity [60]. Figure 8 shows the IS measurements (Nyquist plot) and the corresponding simulations (with the equivalent circuit model presented in Figure 8c) of the OPV cells based on a) PTB7:PC_71_BM and b) PTB7:PC_71_BM:FeS_2_ with 0.5 wt % FeS_2_ at different operating bias values (0.0 V, 0.3 V, 0.6 V and close to *V*_oc_) under illumination. The dark IS measurement is also presented. The fitted lines show good agreement with the measured data. The current IS data are in agreement with previously reported IS data of OPVs based on low band-gap polymers [34,60]. It is possible to get information about the recombination (*R*_rec_) and series resistance (*R*_s_), from the IS curves at low and large frequencies, respectively [34,59–60]. These parameters necessary for the simulation are derived from the experimental data (Figure 8c). More precisely, *R*_s_ is related to the overall resistance of the device influencing the *J*_sc_ value. *R*_rec_ is associated with the charge carrier recombination processes in the device. In general, higher *R*_rec_ values are better for the devices [60]. C is a nonideal capacitor (Figure 8c). Table 2 shows the equivalent circuit parameters (*R*_rec_ and *R*_s_) derived from the fitted data for the PTB7:PC_71_BM and the PTB7:PC_71_BM:FeS_2_-based solar cells. These values are in good agreement with the values reported for the PTB7:PC_71_BM solar cells [34,60]. The *R*_s_ data are similar for both devices, which might suggest that the possible fabrication defects in the stack architecture are comparable in both devices [34]. The *R*_rec_ values rapidly decrease when the applied bias increases from 0.0 V to near *V*_oc_, as shown in Figure 8d. This behavior is due to the fact that at high bias, the carrier density in the OPV increases and recombination takes place more frequently in the device [34,60]. Figure 8d and Table 2 indicate that the *R*_rec_ values are in the same order of magnitude for both systems, but *R*_rec_ is slightly higher for the PTB7:PC_71_BM:FeS_2_ cells (red curve) than for the PTB7:PC_71_BM cells (blue curve). Finally, the IS analysis suggests that electrical losses due to charge carrier recombination are reduced in devices with FeS_2_ NCs (specifically at 0.5 wt %) and, therefore, the *J*_sc_ and FF values (shown in Table 1) of the latter device are improved.

**Table 2:** Parameters of the equivalent circuit used to simulate the experimental IS data of PTB7:PC_71_BM and PTB7:PC_71_BM:FeS_2_ (0.5 wt % FeS_2_) based solar cells.

Device	DC bias (V)	*R*_s_ (Ω·cm^2^)	*R*_rec_ (Ω·cm^2^)

PTB7:PC_71_BM	0.0	1.59	396
	0.3	1.83	155
	0.6	2.22	24
	*V*_oc_	2.20	5.2

PTB7:PC_71_BM:FeS_2_	0.0	1.67	445
(0.5 wt % FeS_2_)	0.3	1.88	177
	0.6	2.32	26
	*V*_oc_	2.27	4.8

**Figure 9:**
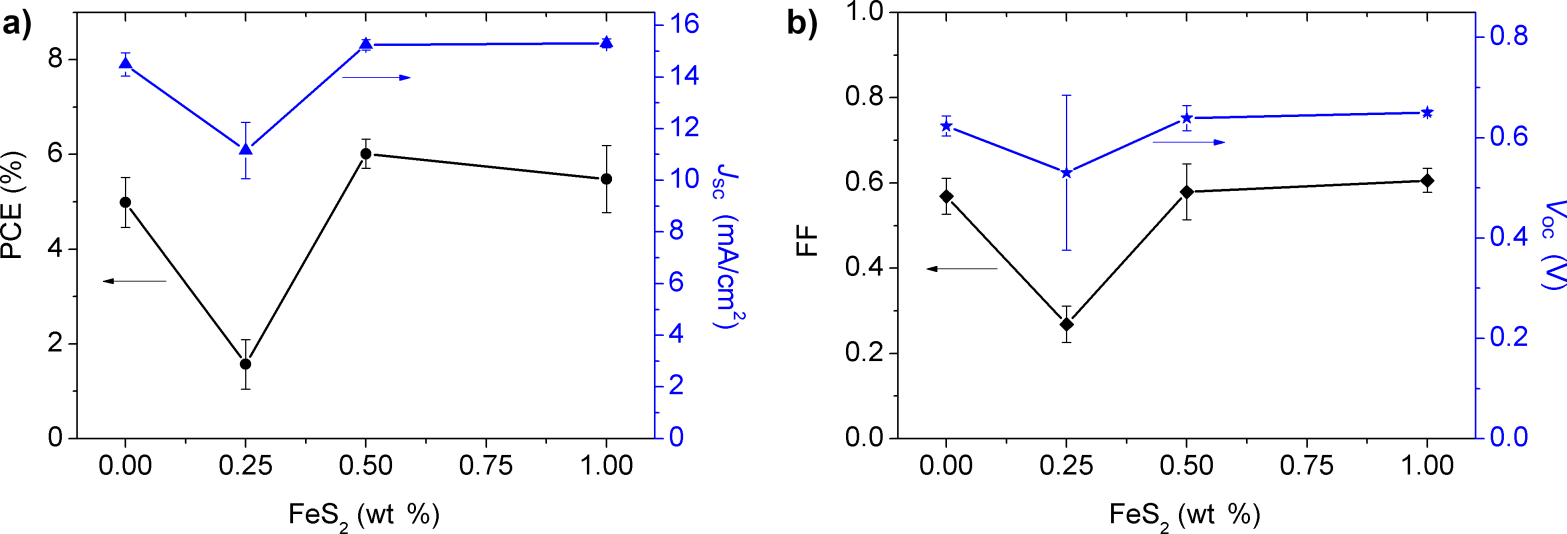
Electrical parameters of the OPVs as a function of FeS_2_ NC content: a) PCE (black curve), *J*_sc_ (blue plot); b) FF (black curve), *V*_oc_ (blue plot). Three different sets and at least three devices for each concentration were tested.

Figure 9 shows the average electrical parameters of the three individual OPV experiments for each concentration as a function of the FeS_2_ content. Compared to the reference OPV (0.0 wt %), the parameters vary only slightly for the OPVs containing 0.5 wt % and 1.0 wt % of FeS_2_ content. However, as previously stated, the PCE is statistically enhanced for the OPV with 0.5 wt % FeS_2_ concentration. In case of the device with 1.0 wt % of FeS_2_, the parameters are somewhat decreased because of the FeS_2_ agglomerates present in the active layer. The electrical parameters decay significantly for the device with 0.25 wt % of FeS_2_, as mentioned before. In summary, by the addition of 0.5 wt % of FeS_2_, better charge generation/transport/collection properties are reached most likely because of the enhanced exciton dissociation and additional charge pathways in the active layer. To the best of our knowledge, this is the first time that FeS_2_ NCs have been added to the PTB7:PC_71_BM active layer, resulting in an improvement of the PCE in the fabricated OPV devices.

The earth-abundant material FeS_2_, has been tested in different photovoltaic areas due to its excellent optoelectronic properties, as previously mentioned. Table 3 depicts a summary of the different usage of iron pyrite in photovoltaic applications. Different structures and sizes of FeS_2_ NPs have been used for various purposes within solar cells, for example, for counter electrodes in DSSCs, for electron acceptors or electron donors in inorganic or hybrid solar cells, as hole transport layers in perovskite solar cells, and as a second electron acceptor in OPVs. For instance, when slightly spherical iron pyrite NPs of 10–25 nm size were used as acceptors in P3HT-based solar cells, very poor PV parameters were attained [10,14–15,61]. When implemented as second electron acceptors in P3HT:PCBM solar cells, the current density increased from 6.69 to 7.63 mA/cm^2^ using a concentration of only 0.3 wt % FeS_2_ with respect to the acceptor [18]. Furthermore, FeS_2_ NPs have been used as a HTL in CdS/CdTe and perovskite solar cells [19–20], among other applications, as shown in Table 3. Consequently, it was interesting to test the effect of FeS_2_ NPs when incorporated into a PTB7:PC_71_BM active layer, as done in this work.

**Table 3:** FeS_2_ structures and influence on the device photovoltaic (PV) parameters.

Morphology	Size (nm)	PV parameters				Use in the PV devices	Ref.
		*V*_oc_ (V)	*J*_sc_ (mA/cm^2^)	FF	PCE (%)		

slightly spherical	10	0.44	0.85	0.42	0.16	acceptor in ITO/PEDOT:PSS/P3HT: FeS_2_/Al	[15]
spherical	10	0.66	7.63	0.47	2.3	second electron acceptor in ITO/PEDOT:PSS/P3HT:PCBM:FeS_2_/Al	[18]
slightly spherical	14.8 ± 3.6	0.41	7 × 10^−3^	0.25	0	acceptor in PEDOT:PSS/P3HT:FeS_2_/Al	[14]
spherical	15	–	–	–	–	donor in ITO/ZnO/FeS_2_ NC/MoO_3_/Au, only photocurrent was obtained	[16]
semispherical	10–100	0.72	0.13	0.55	0.06	acceptor in ITO/PEDOT:PSS/MEHPPV:FeS_2_/Al	[49]
spheroidal single crystal	5–20	–	–	–	–	–	[5]
spherical	15 ± 3	0.71	15.14	0.68	7.31	transparent electrode instead of Pt in DSSC	[12]
quantum dots	10–20	0.58	3.1	0.34	0.61	acceptor in ITO/PEDOT:PSS/P3HT:FeS_2_/ZnO/Ag	[61]
nanoparticles	60	0.16	3.7	0.29	0.5	donor in ITO/PEDOT:PSS/FeS_2_:CdSe/Au	[62]
nanosheets	30–50	0.38	2.04	0.40	0.93	nano films as photocathode in TiO_2_ DSSC	[63]
nanocubes	80	0.79	3.9	0.36	1.1	donor in ITO/PEDOT:PSS/TFB/FeS_2_:CdS/Al	[17]
nanocubes	60–200	–	–	–	–	photoconductor NC film ITO/FeS_2_(400 nm)/Al	[4]
2D nanoplates	200–500	0.78	0.15	–	0.03	acceptor in PEDOT:PSS (40 nm)/P3HT:FeS_2_/Al	[10]
thin film	–	0.43	9.60	0.43	1.76	counter electrode in QDSC TiO_2_/CdS/CdSe/ZnS	[13]
thin film	200 ± 50	0.79	15.20	0.65	7.97	thin film p-type counter electrode in DSSC	[9]
rod-like nanocrystals	17–22		1.301	–	0.45	aceptor in ITO/PEDOT:PSS/ (FeS_2_:PCPDTBT)/Al	[64]
nanocrystals	30 ± 5	0.83	20	0.68	11.6	HTL^a^ in CdS/CdTe cells	[19]
nanocrystals	100	0.94	17.7	0.77	11.2	HTL^a^ in glass/FTO/TiO_2_/perovskite/FeS_2_/Au	[20]

^a^HTL: hole transport layer.

## Conclusion

In this work, FeS_2_ NCs were added as a third component (second electron acceptor) in the PTB7:PC_71_BM active layer of an OPV to enhance the performance of the solar cell. In addition, the influence of the concentration of the FeS_2_ NCs (0, 0.25, 0.5 and 1.0 wt %) on the solar cell performance was investigated. The PV parameters were enhanced at a specific doping level (0.5 wt %). The PCE could be improved by about 21% compared to the reference devices with an average PCE of ≈5% (best = 5.69%), yielding an average PCE of ≈6% (best = 6.47%). Hence, FeS_2_ NCs potentially facilitate charge dissociation, and additional charge-carrier pathways are created and hence, improving charge transport and collection.

## Experimental

### Materials and synthesis of FeS_2_ nanocrystals

All chemicals were used as received without further purification. For FeS_2_ synthesis, iron(II) chloride, octadecylamine and diphenyl ether were acquired from Sigma-Aldrich. For OPV fabrication, indium tin oxide (ITO) covered glass substrates (10 Ω/sq, ≈165 nm thickness) were purchased from Delta Technologies, poly(3,4-ethylene-dioxythiophene):poly(styrenesulfonate) (PEDOT:PSS) (Clevios PVP AI 4083) was acquired from Heraeus and PTB7 and PC_71_BM from 1-Material Inc.

FeS_2_ NCs were prepared using a two-pot method [48]. Iron(II) chloride (0.5 M) and sulfur (0.57 M) precursors were used to obtain the FeS_2_ NCs. The iron precursor was dissolved with octadecylamine at 120 °C for 1 h under argon atmosphere. Sulfur was dissolved with diphenyl ether at 70 °C for 1 h under argon gas. Then sulfur/diphenyl ether solution was added to the iron-octadecylamine complex. The solution was heated and contained at 220 °C for 2 h. Once the reaction was finished, the product was cooled down to 110 °C. Subsequently, 5 mL of chloroform was added, and the solution was kept at room temperature. Finally, 35 mL of methanol was added to purify the product by centrifugation. This step was repeated several times. The final product was dispersed in chloroform for the posterior active layer fabrication.

### Active layer

The solution for the active layer was prepared by dissolving 30 mg/mL of PTB7 and PC_71_BM at 1:1.5 w/w in anhydrous chlorobenzene/1,8-diiodooctane (97:3 v/v) within a glove box under nitrogen atmosphere. The solution was stirred on a hot plate for 24 h at room temperature. FeS_2_ NCs were added to the active layer at concentrations of 0, 0.25, 0.5, and 1.0 wt % with respect to the electron donor and then mixed in an ultrasonic bath for 20 min.

### Glass/ITO/PEDOT:PSS/PTB7:PC_71_BM:FeS_2_/PFN/Field’s metal (FM) fabrication

The OPV devices were fabricated as follows (Figure 1a): Indium tin oxide (ITO) covered glass substrates were cut (≈1.8 cm × 1.8 cm) and ultrasonically cleaned sequentially for about 20 min in a detergent solution, distilled water and ethanol and dried in an oven at 80 °C for at least 12 h. Then, the ITO substrates were treated with UV-ozone plasma for 15 min. A PEDOT:PSS layer of ≈40 nm thickness was spin-coated on top of the ITO substrate at 4500 rpm for 1 min, and then it was thermally treated at 120 °C for 20 min. The active layers with and without FeS_2_ were spin-coated onto ITO/PEDOT:PSS at 1900 rpm for 60 s at atmospheric conditions, and then the films were annealed at 80 °C for 15 min (active layer thickness ≈100 nm). A PFN layer (≈5–10 nm) was spin-coated at 6000 rpm on top of the active layer and exposed to thermal annealing for 15 min at 80 °C. The active area (0.07 cm^2^) was delimited with Scotch tape. The FM top electrode was deposited (after melting it at 95 °C in a hot plate) on top of the PFN layer by drop casting, using the previously reported procedure [34]. The final device structure is as follows: glass (1.1 mm)/ITO (≈165 nm)/PEDOT:PSS (≈40 nm)/PTB7:FeS_2_:PC_71_BM (≈100 nm)/PFN (≈5 nm)/FM. Supporting Information File 1, Figure S1 shows a chart of the device fabrication and characterization procedure.

### Characterization

TEM images were taken with a JEOL JEM-1010 instrument using an acceleration potential of 80 kV and SEM images were acquired with a JEOL JSM 7800F. STM [32–33] measurements were carried out under ambient conditions with the Nanosurf Easyscan 2 STM device. For the latter measurements, FeS_2_ was dissolved in chlorobenzene at a concentration of 0.2 mg/mL and deposited on a highly ordered pyrolytic graphite (HOPG) surface by drop casting. Mechanically cut Pt–Ir wires were used as STM tips. Before the deposition of each film, HOPG substrates were cleaved by using the adhesive tape technique to obtain an atomically clean surface.

Cyclic voltammetry (CV) measurements were carried out using a PARSTAT 2273 potentiostat in a classical three-electrode electrolytic cell. The working electrode was an ITO electrode, the reference electrode was made of Ag^+^/Ag^0^ (0.01 M AgNO_3_/0.1 M tetrabutylammonium perchlorate in acetonitrile) and the counter electrode was made of platinum. All CV measurements were carried out in dry acetonitrile using 0.1 M tetrabutylammonium hexafluorophosphate (TBAPF_6_) as the electrolyte at a scan rate of 100 mVs^−1^, and each solution was purged with N_2_ prior to measurement. UV–vis characterization was performed through thin films of the PTB7:PC_71_BM active layer with and without FeS_2_ NCs at different concentrations (0, 0.25, 0.5 and 0.1 wt %) spin-coated onto a corning glass at 1900 rpm for 60 s in order to obtain similar thickness (≈100 nm). We used a commercial UV–vis spectrometer (Lambda 900, Perkin Elmer Instruments). FTIR spectra were recorded with a Frontier MIR spectrophotometer (Perkin Elmer). The active layer thickness was measured by AFM (Easyscan2 from Nanosurf) in contact mode employing cantilever tips with the aluminum reflective coating (ContAl-G) from BudgetSensors. The AFM roughness images were acquired in dynamic force mode (using PPP-NCLAu from NanoSensors), because it shows better resolution than the contact mode [65]. For these AFM measurements, sample films were prepared under the same OPV fabrication procedure (without PFN neither FM). OPVs were illuminated with the Air Mass 1.5 spectrum provided by a solar simulator Sciencetech SS150 class AAA that was calibrated using an Oriel reference cell. Current density versus voltage (*J*–*V*) curves were measured using a Keithley 2450 source meter under ambient conditions. External quantum efficiency (EQE or IPCE) was measured in a home-built EQE set up [32]. A potentiostat/galvanostat PARTAT 2273 system was used for the IS measurements. The impedance spectra were measured at ambient atmosphere and at room temperature under dark and illumination conditions using a frequency range from 1 to 500 kHz with an amplitude of 20 mV. The curves were simulated with the ZView software [34].

## Supporting Information

File 1 Statistical tests and SEM images.
