# Supplementary material for: Nontoxic pyrite iron sulfide nanocrystals as second electron acceptor in PTB7:PC71BM-based organic photovoltaic cells
Source: Beilstein J Nanotechnol. 2019 Nov 14;10:2238–50. doi: 10.3762/bjnano.10.216 (PMC6880843; doi:10.3762/bjnano.10.216)
Supplement: File 1 — Statistical tests and SEM images. [file Beilstein_J_Nanotechnol-10-2238-s001.pdf]

BEILSTEIN JOURNAL OF NANOTECHNOLOGY

Supporting Information

for

Nontoxic pyrite iron sulfide nanocrystals as second electron acceptor in PTB7:PC_{71}BM-based organic photovoltaic cells

Olivia Amargós-Reyes, José-Luis Maldonado, Omar Martínez-Alvarez, María-Elena Nicho, José Santos-Cruz, Juan Nicasio-Collazo, Irving Caballero-Quintana and Concepción Arenas-Arrocena

Beilstein J. Nanotechnol. **2019,** *10,* 2238–2250. doi:10.3762/bjnano.10.216

Statistical tests and SEM images

License and Terms: This is a supporting information file under the terms of the Creative Commons Attribution License (http://creativecommons.org/licenses/by/4.0). Please note that the reuse, redistribution and reproduction in particular requires that the authors and source are credited.
The license is subject to the *Beilstein Journal of Nanotechnology* terms and conditions: (https://www.beilstein-journals.org/bjnano)

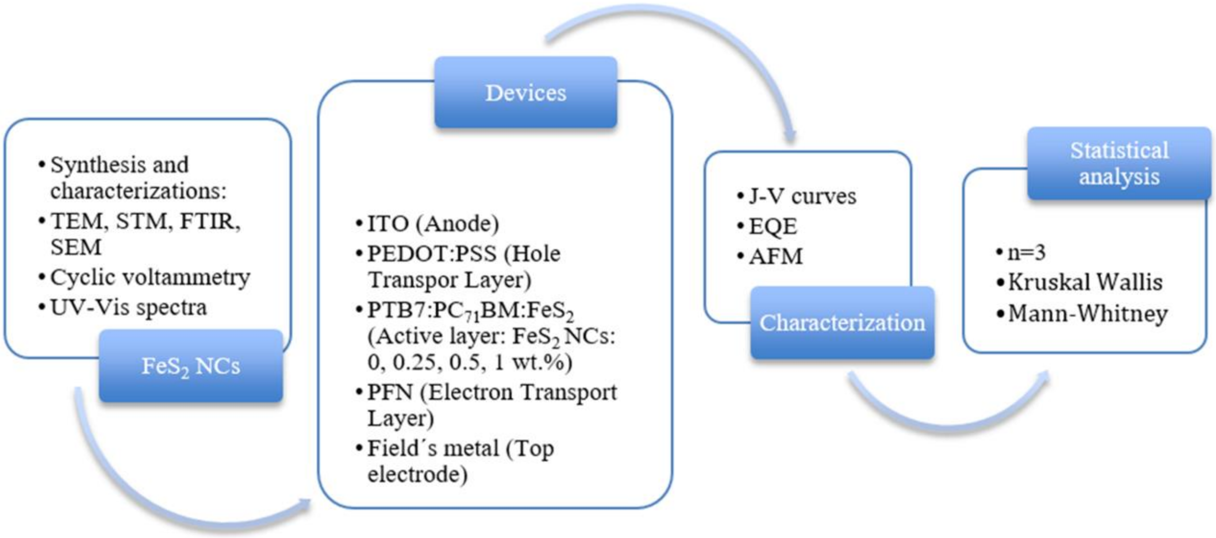

Figure S1: Chart of the experimental procedure and OPVs fabrication and characterization.

Statistical tests

Table S1: Kruskal-Wallis Test. Significant difference between groups.

	V_{oc}	**FF**	J_{sc}	**PCE**
**Z Value**	10.362	22.859	10.393	23.707
**º l**	3	3	3	3
P value	**0.016**	**<0.0001**	**0.016**	**<0.0001**

Table S2: Mann-Whitney test. Comparison between groups.

**Groups based on FeS_2 (wt.%)**		V_{oc}	**FF**	J_{sc}	**PCE**
**0 vs 0.25**	Z	-2.910	-3.909	-.301	-3.907
	p *Value*	**.004**	**.0001**	.764	**.0001**
**0 vs 0.5**	Z	-.664	-2.331	-2.296	-2.419
	p *Value*	.507	**.020**	**.022**	**.016**
**0 vs 1.0**	Z	-.494	-1.539	-1.668	-2.018
	p *Value*	.622	.124	.095	**.044**
**0.25 vs 0.5**	Z	-2.424	-3.643	-2.939	-3.674
	p *Value*	**.015**	**.0001**	**.003**	**.0001**

**0.25 vs 1.0**	Z	-2.370	-2.900	-1.886	-2.831
	p *Value*	**.018**	**.004**	.059	**.005**
**0.5 vs 1.0**	Z	-.110	-.219	-.380	-.163
	p *Value*	.912	.827	.704	.870

Bold letters indicate statistical significance.

SEM images

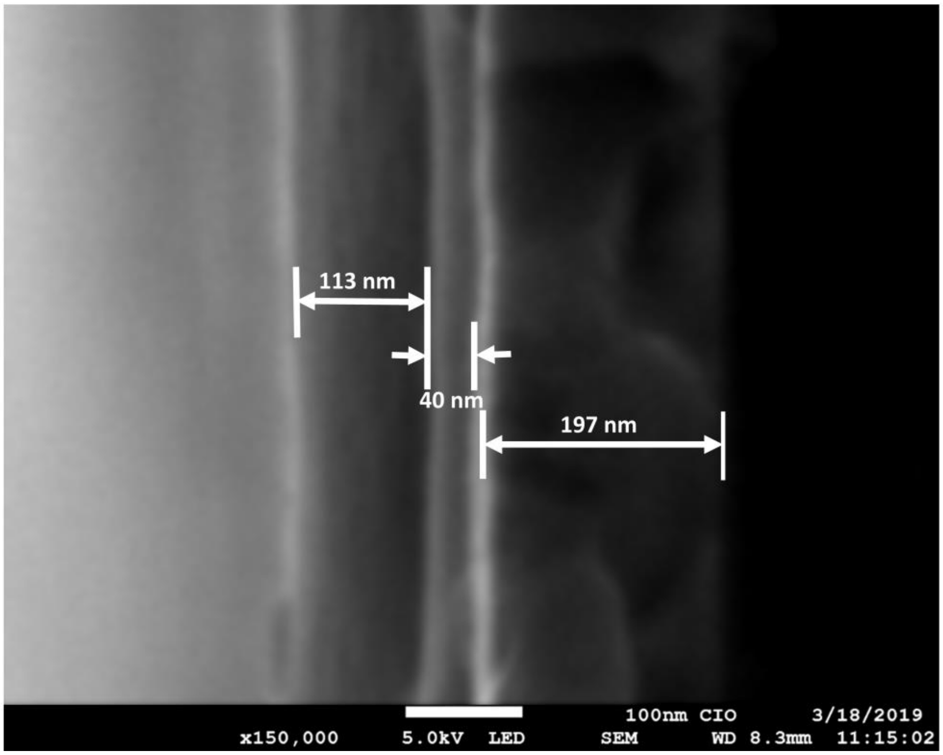

Figure S2: SEM image of OPVs layers (ITO ~197 nm; PEDOT:PSS ~40 nm; PTB7:$PC_{71}BM$ ~113 nm).

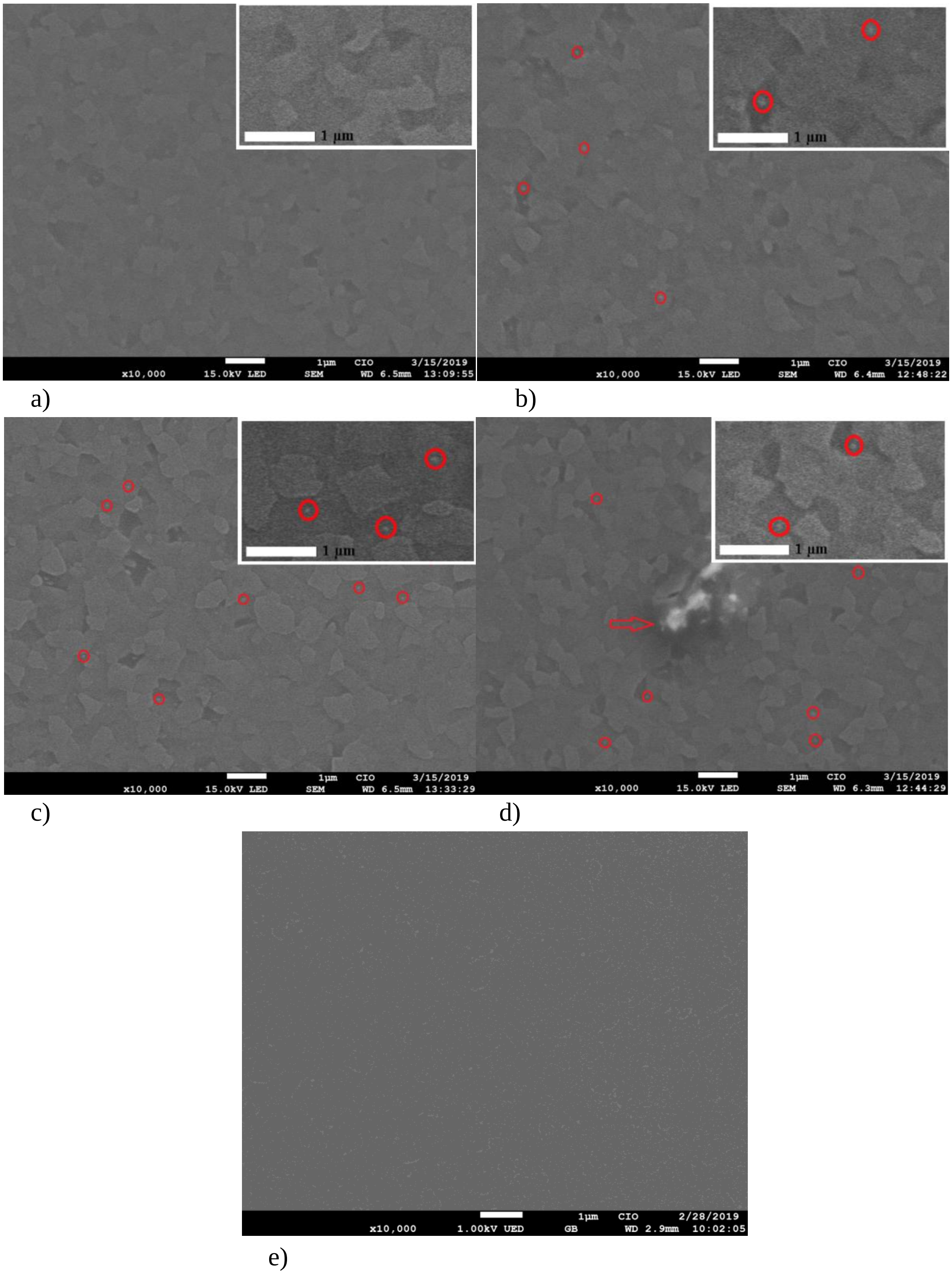

Figure S3: SEM images (at 15 kV) of OPVs active layer with different concentrations of FeS_2: a) 0.0 wt %, b) 0.25 wt %, c) 0.5 wt % and d) 1.0 wt %; e) image of PEDOT:PSS at a lower SEM voltage (1 kV). Scale bar = 1 µm.
